# Long noncoding RNA SNHG5 promotes podocyte injury *via* the microRNA-26a-5p/TRPC6 pathway in diabetic nephropathy

**DOI:** 10.1016/j.jbc.2022.102605

**Published:** 2022-10-17

**Authors:** Yan Zhou, Zuo-Lin Li, Lin Ding, Xing-Jian Zhang, Nan-Chi Liu, Shan-Shan Liu, Yan-Fei Wang, Rui-Xia Ma

**Affiliations:** 1 Department of Nephrology, Affiliated Hospital of Qingdao University, Qingdao, Shandong, China; 2 Institute of Nephrology, Zhong Da Hospital, Southeast University School of Medicine, Nanjing, Jiangsu, China

**Keywords:** diabetic nephropathy, podocyte injury, lncRNA SNHG5, miR-26a-5p, TRPC6, ACR, albumin to creatinine ratio, DM, diabetes mellitus, DN, diabetic nephropathy, FISH, fluorescence *in situ* hybridization, HG, high glucose, HM, high mannitol, lncRNA, long noncoding RNA, miRNA, microRNAs, PAS, periodic acid schiff, RIP, RNA immunoprecipitation, SNHG5, small nucleolar RNA host gene 5, TRPC6, transient receptor potential canonical type 6, TEM, transmission electron microscope

## Abstract

Podocyte injury is a characteristic pathological hallmark of diabetic nephropathy (DN). However, the exact mechanism of podocyte injury in DN is incompletely understood. This study was conducted using db/db mice and immortalized mouse podocytes. High-throughput sequencing was used to identify the differentially expressed long noncoding RNAs in kidney of db/db mice. The lentiviral shRNA directed against long noncoding RNA small nucleolar RNA host gene 5 (SNHG5) or microRNA-26a-5p (miR-26a-5p) agomir was used to treat db/db mice to regulate the SNHG5/miR-26a-5p pathway. Here, we found that the expression of transient receptor potential canonical type 6 (TRPC6) was significantly increased in injured podocytes under the condition of DN, which was associated with markedly decreased miR-26a-5p. We determined that miR-26a-5p overexpression ameliorated podocyte injury in DN *via* binding to 3′-UTR of *Trpc6*, as evidenced by the markedly reduced activity of luciferase reporters by miR-26a-5p mimic. Then, the upregulated SNHG5 in podocytes and kidney in DN was identified, and it was proved to sponge to miR-26a-5p directly using luciferase activity, RNA immunoprecipitation, and RNA pull-down assay. Knockdown of SNHG5 attenuated podocyte injury *in vitro*, accompanied by an increased expression of miR-26a-5p and decreased expression of TRPC6, demonstrating that SNHG5 promoted podocyte injury by controlling the miR-26a-5p/TRPC6 pathway. Moreover, knockdown of SNHG5 protects against podocyte injury and progression of DN *in vivo*. In conclusion, SNHG5 promotes podocyte injury *via* the miR-26a-5p/TRPC6 pathway in DN. Our findings provide novel insights into the pathophysiology of podocyte injury and a potential new therapeutic strategy for DN.

Diabetes mellitus (DM) is a global public health problem (1). Diabetic nephropathy (DN) is among the most common and severe microvascular complications of DM and the primary cause of end-stage renal disease. Glomerular hypertrophy, podocyte injury, glomerular basement membrane thickening, and extracellular matrix deposition are characteristic pathological features of DN (2), and podocyte injury is thought to be a central link in the occurrence of DN. Nevertheless, the precise mechanisms of podocyte injury in DN are incompletely understood.

Transient receptor potential canonical type 6 (TRPC6) is a nonselective receptor-operated cation channel that regulates reactive fibrosis and growth signaling (3). Our previous studies found that TRPC6 is significantly upregulated in injured podocytes in the context of DN and plays a significant role in podocyte injury. Mechanistically, upregulated TRPC6 promotes podocyte injury by activating the NFAT pathway (4, 5). Targeting TRPC6 using pharmacological or genetic interventions could ameliorate podocyte injury and delay the progression of kidney diseases (6, 7). Nevertheless, the underlying mechanisms of dysregulated expression of TRPC6 in DN remain obscure.

MicroRNAs (miRNAs) are a class of small noncoding RNAs that play essential regulatory roles in almost all biological processes by modulating gene expression (8). Several lines of evidence suggest that miRNAs play crucial roles in the occurrence and development of kidney diseases (9). In the context of DN, associations between podocyte injury and miRNA expression levels in the blood, urine, and kidney tissue have been reported (10). Moreover, modulation of the expression of several miRNAs has been shown to protect against podocyte injury in DN (10). Nevertheless, it is uncertain whether specific miRNAs regulate TRPC6 in DN.

Long noncoding RNAs (lncRNAs), commonly defined as RNA molecules more than 200 nucleotides in length, are significant players at almost every level of gene function and regulation (11). Several studies reported that lncRNAs combine with miRNAs such that the targeted mRNA can escape negative regulation by a mechanism known as competing endogenous RNA (12), and lncRNA appears to participate in diabetes and DN (13). For instance, Deng *et al.* (14) found that increased lncRNA Meg3 expression contributed to podocyte injury induced by high glucose, suggesting that specific lncRNA plays a critical role in the progression of DN. However, regulation of podocyte injury by specific lncRNAs *via* the TRPC6 pathway has not been reported.

## Results

### TRPC6 expression increases significantly in injured podocytes in DN

**Figure 1:**
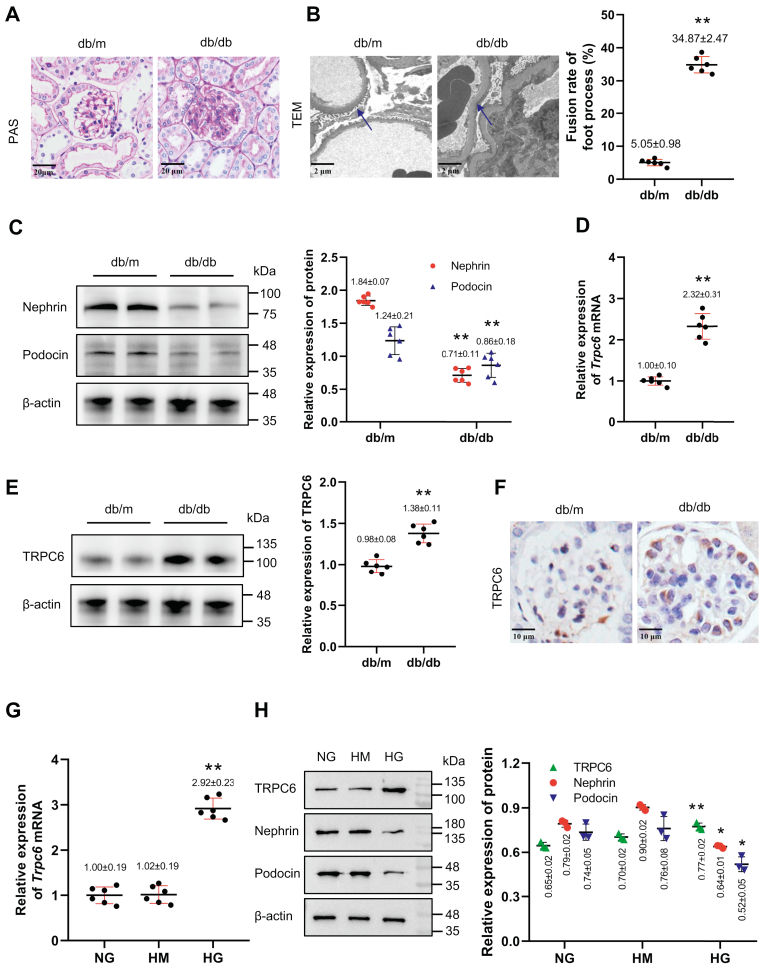
**Increased expression of TRPC6 in injured podocytes under DN conditions.***A*, histological changes in glomeruli of db/m and db/db mice were shown by PAS staining (magnifications ×400). *B*, the ultrastructure of kidney in db/m and db/db mice for transmission electron microscopy (*left panel*, TEM, magnifications ×8000) and fusion rate of foot process in mice (*right panel*, n = 6), the *blue arrow* indicates foot process. ∗∗ *p* < 0.01 *versus* db/m (Student's *t* test). *C*, protein levels of nephrin and podocin normalized to β-actin in kidney (Western blot, n = 6). ∗∗ *p* < 0.01 *versus* db/m (Student's *t* test). *D* and *E*, the level of *Trpc6* mRNA (*D*) and TRPC6 protein (*E*) normalized to β-actin in kidney were measured by qPCR and Western blot, respectively (n = 6). ∗∗ *p* < 0.01 *versus* db/m (Student's *t* test). (*C*) and (*E*) shared the same blotting sheet and reference protein. *F*, the distribution of TRPC6 in glomeruli of db/m and db/db mice shown by immunohistochemistry (magnifications ×600). *G* and *H*, levels of *Trpc6* mRNA (*G*) and TRPC6, nephrin, and podocin protein (*H*) (all normalized to β-actin) in podocytes exposed to NG (5.5 mM glucose), HG (30 mM glucose), and HM (5.5 mM glucose+ 24.5 mannitol) measured by qPCR (n = 6) and Western blot (n = 3), respectively. ∗ *p* < 0.05 *versus* NG, ∗∗ *p* < 0.01 *versus* NG (ANOVA followed by Bonferroni correction). All data above are represented as means ± standard deviation. HG, high glucose; HM, high mannitol; NG, normal glucose; PAS, periodic acid Schiff; TEM, transmission electron microscope; TRPC6, transient receptor potential canonical type 6.

The baseline indicators of db/db mice are shown in Table 1. Histologic analysis revealed marked glomerular hypertrophy and mesangial expansion in db/db mice compared with control db/m mice (Fig. 1*A*). Transmission electron microscope (TEM) revealed that the foot processes were fused, and basement membranes were thickened in db/db mice (Fig. 1*B*). The expression of nephrin and podocin was decreased significantly (Fig. 1*C*), suggesting podocyte injury. Then, we found that *Trpc6* mRNA and protein levels were increased significantly in the kidneys of db/db mice (Fig. 1, *D* and *E*). Immunostaining of TRPC6 indicated that the TRPC6 expression significantly increased in injured podocytes (Fig. 1*F*). Podocyte injury and upregulated TRPC6 expression were also observed in high glucose (HG)-treated podocytes, detected by qPCR (Fig. 1*G*) and Western blot (Fig. 1*H*), respectively. These findings suggest that TRPC6 expression increases significantly in injured podocytes in DN.

**Table 1:** Baseline indicators of mice ($\bar{x}$ ± s)

Indicators	db/m (n = 6)	db/db (n = 6)	*t* value	*p* value
KW/BW (mg/g)	12.51 ± 2.61	9.31 ± 1.06	2.778	0.020
FBG (mmol/L)	5.32 ± 0.41	17.54 ± 2.29	12.836	0.000
ACR (mg/mg)	0.24 ± 0.12	1.09 ± 0.41	4.886	0.001
TG (mmol/L)	1.50 ± 0.29	3.25 ± 0.22	11.696	0.000
TC (mmol/L)	2.91 ± 0.44	5.41 ± 0.86	6.362	0.000
LDL-C (mmol/L)	0.77 ± 0.90	1.23 ± 0.15	6.590	0.000
Scr (μmol/L)	13.06 ± 0.80	13.40 ± 1.51	0.489	0.636
BUN (mmol/L)	8.40 ± 1.03	8.16 ± 0.91	0.435	0.673

Abbreviations: ACR, urinary albumin to creatinine ratio; BUN, serum urea nitrogen; FBG, fasting blood glucose; KW/BW, kidney weight/body weight; LDL-C, low density lipoprotein-cholesterol; Scr, serum creatinine; TC, total cholesterol; TG, triglycerides.

### miR-26a-5p is downregulated and targets TRPC6 in injured podocytes

**Figure 2:**
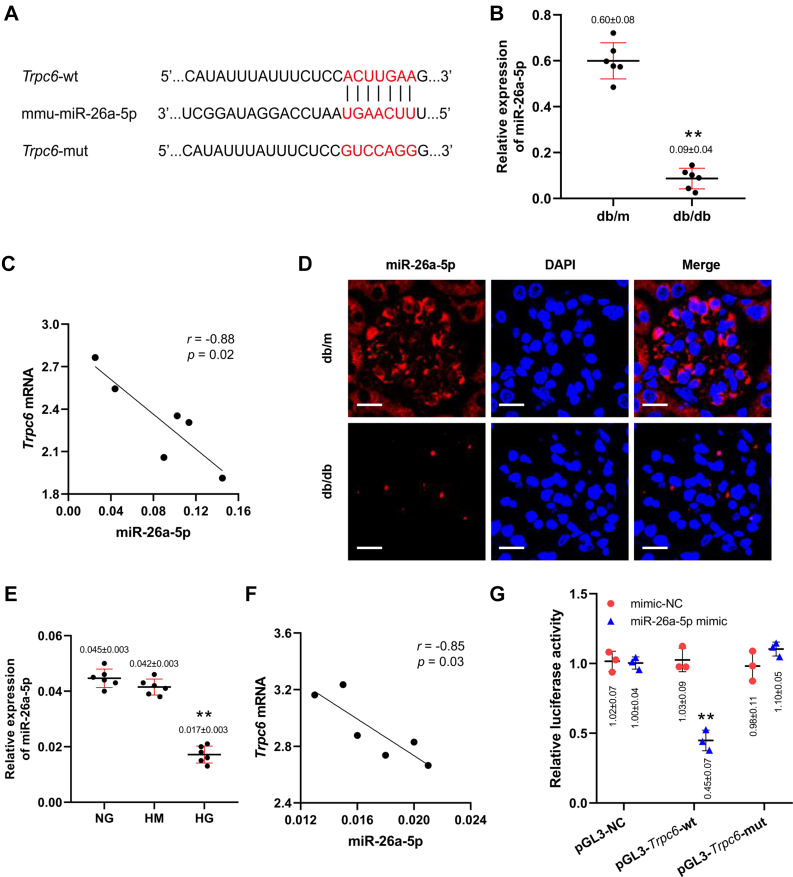
**MiR-26a-5p is downregulated and targets TRPC6 in injured podocytes.***A*, the predicted binding sites between miR-26a-5p and *Trpc6* 3′-UTR from online databases. *B*, the miR-26a-5p level normalized to U6 in kidney of db/m and db/db mice were detected by qPCR (n = 6). ∗∗*p* < 0.01 *versus* db/m (Student's *t* test). *C*, the relationship between miR-26a-5p and *Trpc6* mRNA in kidney evaluated by *Pearson* analysis (n = 6). *D*, the miR-26a-5p expression in glomeruli of db/m and db/db mice shown by FISH (magnifications ×600), scale bars = 10 μm. *E*, levels of miR-26a-5p normalized to U6 in podocytes exposed to NG, HG, and HM (qPCR, n = 6). ∗∗*p* < 0.01 *versus* NG (ANOVA followed by Bonferroni correction). *F*, the relationship between miR-26a-5p and *Trpc6* mRNA levels in HG-treated podocytes were evaluated by *Pearson* analysis (n = 6). *G*, luciferase reporter activity in podocytes cotransfected with *Trpc6*-wt or *Trpc6*-mut and miR-26a-5p mimic or mimic-NC, ∗∗*p* < 0.01 *versus* pGL3-NC (ANOVA followed by Bonferroni correction) and mimic-NC (Student's *t* test). All data above are represented as means ± standard deviation. FISH, fluorescence *in situ* hybridization; HG, high glucose; HM, high mannitol; NG, normal glucose; TRPC6, transient receptor potential canonical type 6.

Given the crucial role of miRNAs in the progression of kidney diseases, we hypothesized that specific miRNAs might mediate dysregulated TRPC6 expression. We predicted the putative miRNAs containing the binding sites of *Trpc6* that contribute to the podocyte injury using five online databases: TargetScan, miRDB, miRanda, DIANA-TarBase, and PicTar. Overlap analysis revealed that miR-26a-5p contains a highly conserved consequence targeting the *Trpc6* 3′-UTR (Fig. 2*A*). We also observed that levels of miR-26a-5p in kidney tissue were markedly decreased (Fig. 2*B*), negatively correlated with *Trpc6* mRNA levels (Fig. 2*C*). Fluorescence *in situ* hybridization (FISH) analysis revealed that the decreased miR-26a-5p was located in the podocytes of glomeruli (Fig. 2*D*).

We then measured expression levels of miR-26a-5p in podocytes exposed to HG. As expected, miR-26a-5p levels decreased significantly (Fig. 2*E*). Consistent with the *in vivo* finding, there is a negative correlation between miR-26a-5p and *Trpc6* mRNA levels in HG-treated podocytes (Fig. 2*F*). The luciferase reporter assay showed that the activity of luciferase reporters was markedly reduced by miR-26a-5p mimic compared with mimic-NC. Furthermore, the activity of *Trpc6* 3′-UTR-mut luciferase reporter was not affected by the miR-26a-5p mimic, suggesting that miR-26a-5p directly interacts with 3′-UTR of *Trpc6* mRNA. (Fig. 2*G*). These findings suggest that miR-26a-5p is involved in podocyte injury by targeting *Trpc6* mRNA directly.

### miR-26a-5p overexpression ameliorates podocytes injury by targeting TRPC6 in DN

**Figure 3:**
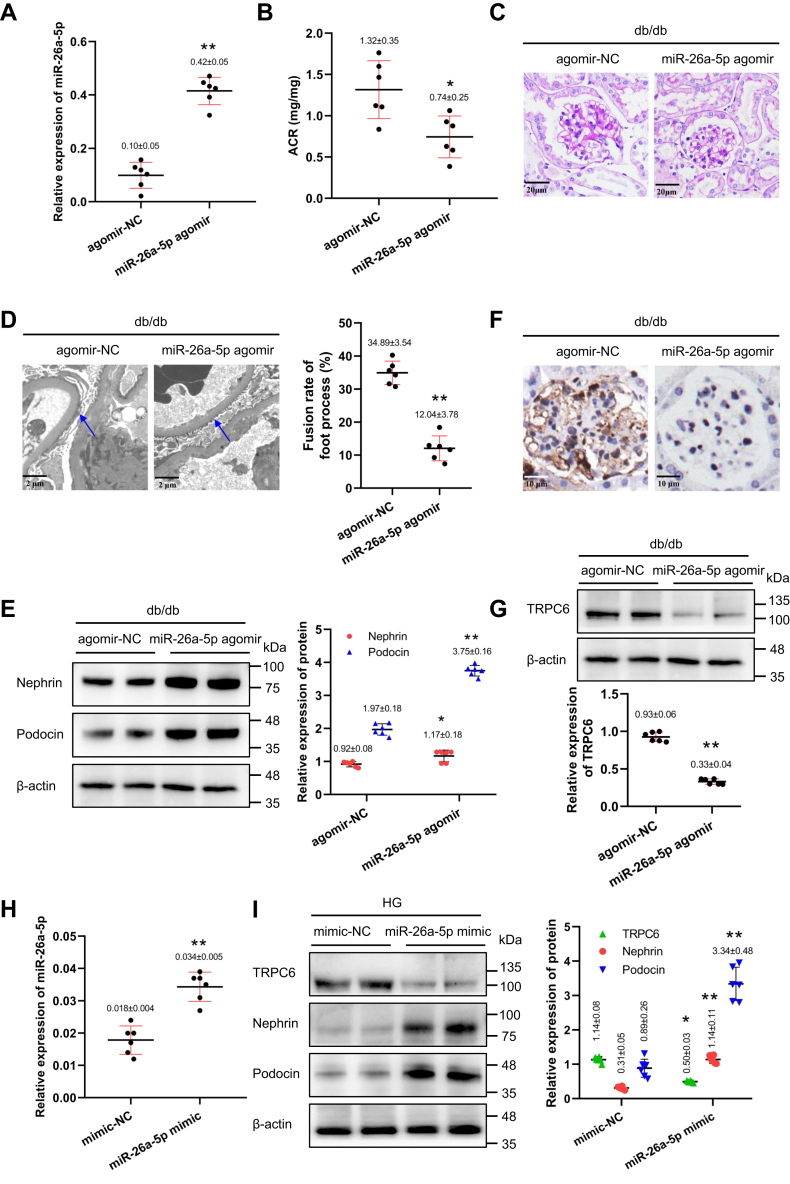
**MiR-26a-5p overexpression ameliorates podocyte injury by targeting TRPC6 in DN.***A*, levels of miR-26a-5p normalized to U6 in kidney of db/db mice after miR-26a-5p overexpression (qPCR, n = 6), ∗∗*p* < 0.01 *versus* agomir-NC (Student's *t* test). *B*, ACR measured after miR-26a-5p overexpression (n = 6), ∗*p* < 0.05 *versus* agomir-NC (Student's *t* test), ACR: urinary albumin to creatinine ratio. *C*, histological changes in glomeruli of db/db mice after miR-26a-5p overexpression shown by PAS staining (magnifications ×400). *D*, the ultrastructure of kidney for TEM (*left panel*, magnifications ×8000) and fusion rate of foot process in mice (*right panel*, n = 6), the *blue arrow* indicates foot process. ∗∗*p* < 0.01 *versus* agomir-NC (Student's *t* test). *E*, the protein levels of nephrin and podocin relative to control in kidney of db/db mice after miR-26a-5p overexpression (Western blot, n = 6). ∗*p* < 0.05 *versus* agomir-NC and ∗∗*p* < 0.01 *versus* agomir-NC (Student's *t* test). *F*, the immunohistochemistry for TRPC6 in glomeruli of db/db mice after miR-26a-5p overexpression (magnifications ×600). *G*, the protein level of TRPC6 relative to control in kidney of db/db mice after miR-26a-5p overexpression (Western blot, n = 6). ∗∗ *p* < 0.01 *versus* agomir-NC (Student's *t* test). (*E*) and (*G*) shared the same blotting sheet and reference protein. *H*, the expression of miR-26a-5p normalized to U6 in HG-treated podocytes after overexpression (qPCR, n = 6), ∗∗ *p* < 0.01 *versus* mimic-NC (Student's *t* test). *I*, the protein levels of TRPC6, nephrin, and podocin relative to control in HG-treated podocytes (Western blot, n = 6), ∗*p* < 0.05 *versus* mimic-NC and ∗∗*p* < 0.01 *versus* mimic-NC (Student's *t* test). All data above are represented as means ± standard deviation. DN, diabetic nephropathy; HG, high glucose; HG, high glucose; PAS, periodic acid Schiff; TEM, transmission electron microscope; TRPC6, transient receptor potential canonical type 6.

To determine the role of miR-26a-5p in podocyte injury and progression of DN *in vivo*, miR-26a-5p agomir or negative control was administered *via* the tail vein of db/db mice. We first confirmed transfection efficiency (Fig. 3*A*). The level of albuminuria was markedly decreased in miR-26a-5p agomir-treated mice (Fig. 3*B*). Histologic analysis revealed that glomerular hypertrophy and mesangial expansion were significantly blunted in miR-26a-5p agomir-treated mice compared with agomir NC-treated mice (Fig. 3*C*). TEM revealed that polysaccharide aggregation and foot process fusion were partially relieved in miR-26a-5p agomir-transfected db/db mice (Fig. 3*D*). Podocyte injury was markedly alleviated, as demonstrated by nephrin and podocin expression levels (Fig. 3*E*). TRPC6 expression was decreased in this group (Fig. 3, *F* and *G*).

In an *in vitro* experiment, podocytes were transfected with miR-26a-5p mimic to overexpress miR-26a-5p before intervention with HG (Fig. 3*H*). As expected, the expression of TRPC6 was markedly decreased in podocytes with miR-26a-5p mimic treatment, combined with the increased nephrin and podocin expression (Fig. 3*I*). These findings suggest that miR-26a-5p overexpression mitigated podocyte injury by inhibiting TRPC6.

### Increased lncRNA small nucleolar RNA host gene 5 expression occurs in injured podocytes in DN

**Figure 4:**
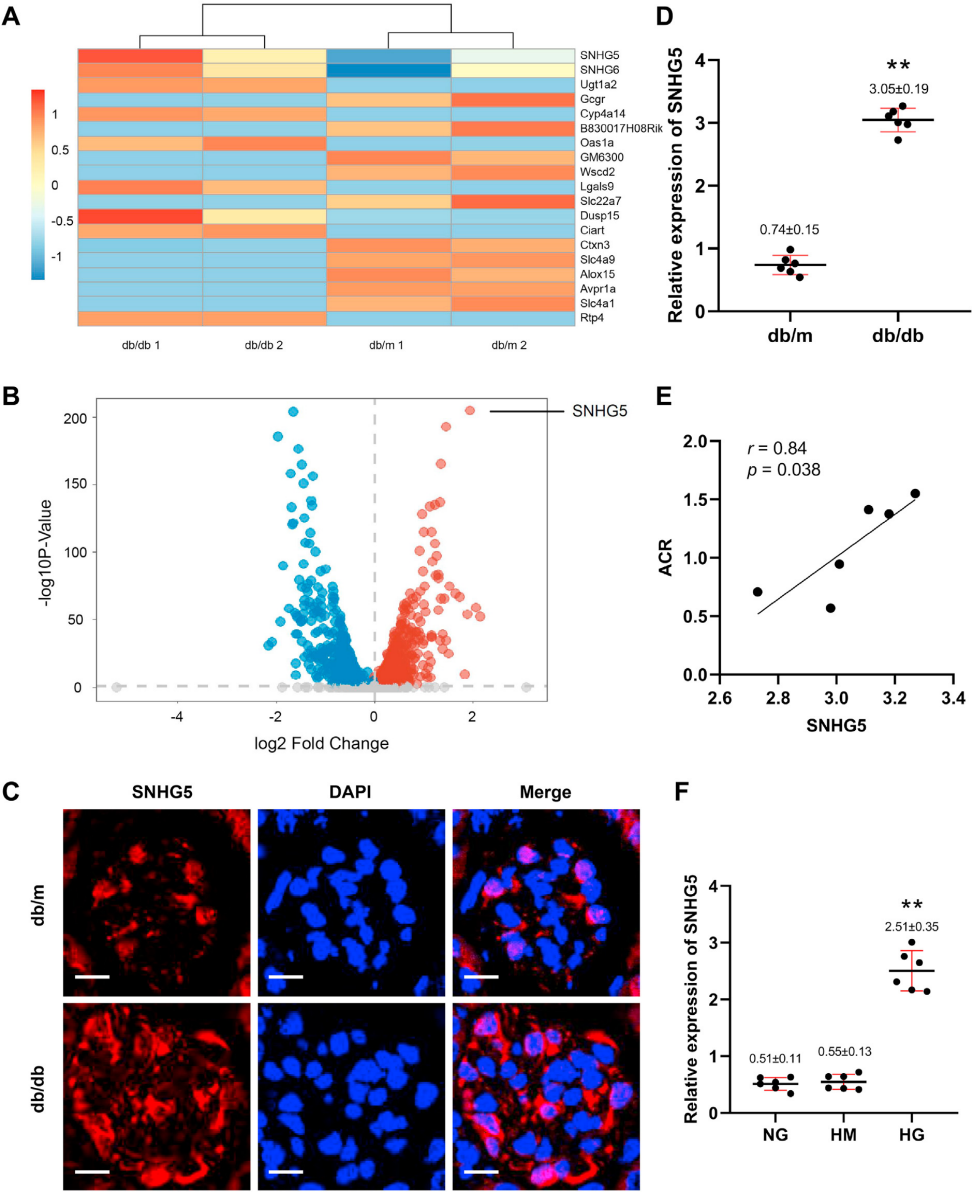
**LncRNA SNHG5 expression increases in injured podocytes under DN condition.** Heat map (*A*) and volcano map (*B*) demonstrating lncRNA expression in mouse kidneys using high-throughput sequencing. *C*, the expression of SNHG5 in glomeruli of db/m and db/db mice shown by FISH (magnifications ×600), scale bars = 10 μm. *D*, the expression level of SNHG5 normalized to β-actin in kidneys of db/m and db/db mice (n = 6). ∗∗*p* < 0.01 *versus* db/m (Student's *t* test). *E*, the relationship between SNHG5 level and ACR evaluated by *Pearson* analysis. ACR: urinary albumin to creatinine ratio. *F*, SNHG5 level normalized to β-actin in podocytes exposed to NG, HG, and HM (n = 6), ∗∗*p* < 0.01 *versus* NG (ANOVA followed by Bonferroni correction). All data above are represented as means ± standard deviation. DN, diabetic nephropathy; FISH, fluorescence *in situ* hybridization; HG, high glucose; HM, high mannitol; lncRNA, long noncoding RNA; NG, normal glucose; SNHG5, small nucleolar RNA host gene 5.

To explore the potential mechanism of the dysregulated miR-26a-5p/TRPC6 pathway, high-throughput sequencing for lncRNAs of the kidney tissue of db/db mice was used (Fig. 4, *A* and *B*). Among the top lncRNAs, the specific lncRNAs for miR-26a-5p interaction were predicted by the StarBase database. A significant increase in lncRNA small nucleolar RNA host gene 5 (SNHG5) was found, and it was selected for further study. To confirm the lncRNA profile, we measured expression levels of SNHG5 *via* FISH and qPCR. FISH revealed that SNHG5 expression was significantly increased, primarily in podocyte cytoplasm (Fig. 4*C*). We found that SNHG5 expression was upregulated in the kidney of db/db mice using qPCR (Fig. 4*D*), positively correlated with the albumin to creatinine ratio (ACR) (Fig. 4*E*). Dramatically increased SNHG5 expression was observed in HG-treated podocytes compared with normal glucose (NG)-treated and high mannitol (HM)-treated podocytes (Fig. 4*F*). These findings suggest that increased SNHG5 expression may be associated with podocyte injury in DN.

### LncRNA SNHG5 promotes podocyte injury via targeting miR-26a-5p

**Figure 5:**
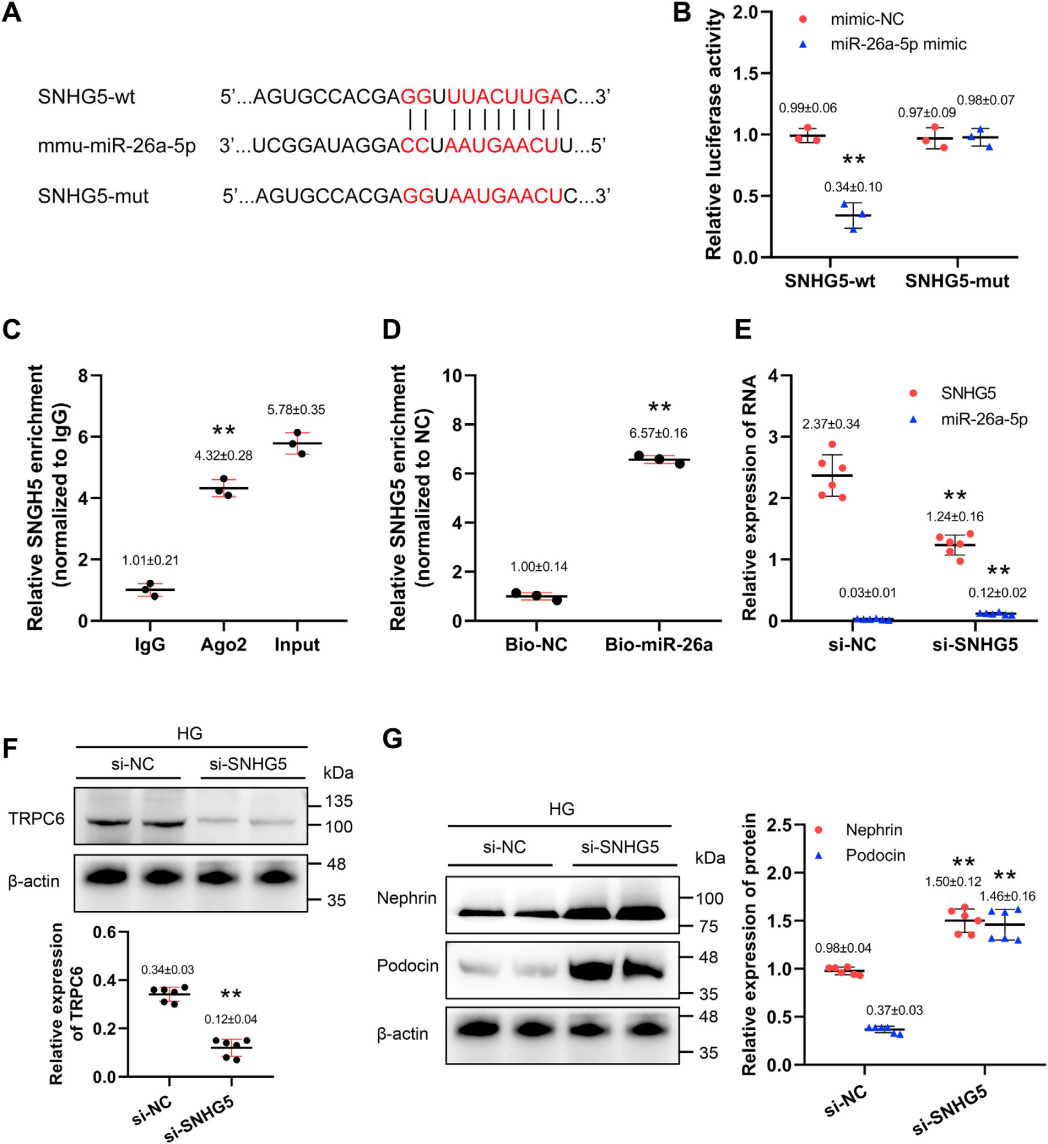
**SNHG5 promotes podocyte injury by targeting miR-26a-5p.***A*, the predicted binding sites between SNHG5 and miR-26a-5p from online databases. *B*, luciferase reporter activity in podocytes cotransfected with SNHG5-wt or SNHG5-mut and miR-26a-5p mimic or mimic-NC, ∗∗*p* < 0.01 *versus* mimic-NC (Student's *t* test). *C*, RIP assays using Ago2 antibody or control IgG antibody in podocytes; qPCR analysis to measure the enrichment of SNHG5 co-precipitated RNA, ∗∗*p* < 0.01 *versus* IgG (Student's *t* test). *D*, RNA pull-down assay to determine the binding between SNHG5 and miR-26a-5p in podocytes, ∗∗*p* < 0.01 *versus* Bio-NC (Student's *t* test). *E*, SNHG5 and miR-26a-5p levels in HG-treated podocytes after SNHG5 knockdown (qPCR, n = 6), ∗∗ *p* < 0.01 *versus* si-NC (Student's *t* test). *F* and *G*, protein levels of TRPC6 (*F*), nephrin and podocin (*G*) normalized to β-actin in HG-treated podocytes after SNHG5 knockdown (Western blot, n = 6), ∗∗*p* < 0.01 *versus* mimic-NC (Student's *t* test). (*F*) and (*G*) shared the same blotting sheet and reference protein. All data above are represented as means ± standard deviation. HG, high glucose; RIP, RNA immunoprecipitation; SNHG5, small nucleolar RNA host gene 5; TRPC6, transient receptor potential canonical type 6.

Next, we sought to understand how SNHG5 promotes podocytes injury. Based on bioinformatics analysis, miR-26a-5p was predicted to contain appropriate binding sites for SNHG5 (Fig. 5*A*), a critical negative regulator of the TRPC6 signaling pathway. We performed, dual-luciferase reporter assay, RNA immunoprecipitation (RIP) and RNA pull-down to test whether there is a direct interaction between SNHG5 and miR-26a-5p in podocytes. Luciferase activity of SNHG5-wt reporter was significantly decreased with miR-26a-5p overexpression, whereas no significant change was observed in SNHG5-mut reporter expression (Fig. 5*B*). In RIP assays, SNHG5 levels were enriched in abundance by Ago2 antibody compared with control IgG (Fig. 5*C*). RNA pull-down analysis displayed a substantial enrichment of SNHG5 by miR-26a-5p compared with the control group (Fig. 5*D*). We found that miR-26a-5p levels were significantly increased in podocytes with SNHG5 knockdown (Fig. 5*E*), combined with decreased TRPC6 expression (Fig. 5*F*). Podocyte injury was markedly abrogated in this group (Fig. 5*G*). These findings suggest that SNHG5 promotes podocyte injury by targeting miR-26a-5p in DN.

### LncRNA SNHG5 knockdown attenuates podocyte injury *in vivo*

**Figure 6:**
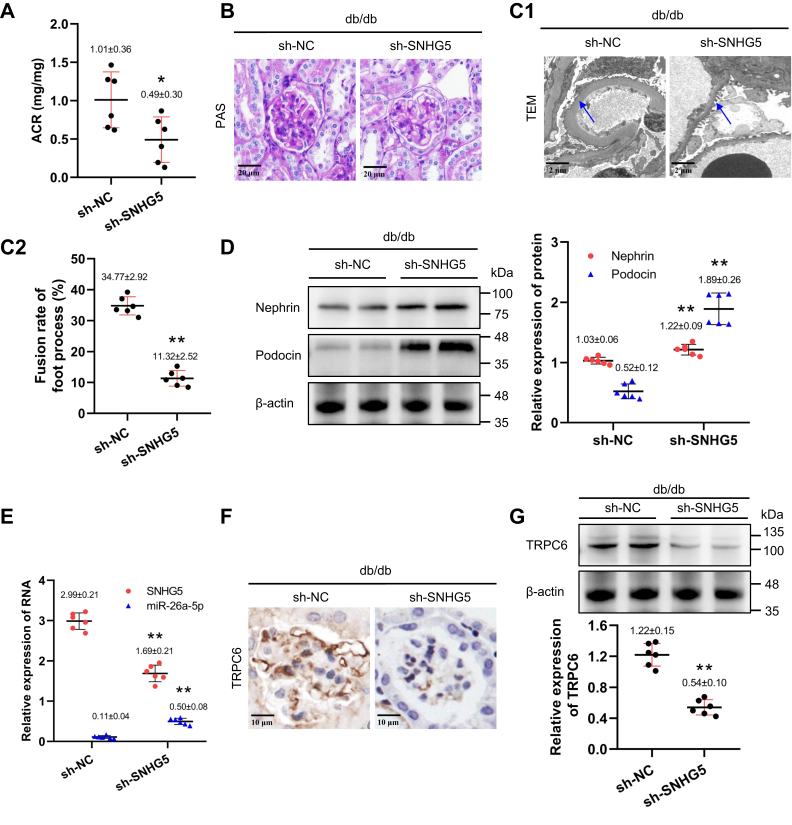
**SNHG5 knockdown attenuates podocyte injury *in vivo*.***A*, ACR of db/db mice measured after SNHG5 knockdown (n = 6); ∗ *p* < 0.05 *versus* sh-NC (Student's *t* test); ACR: urinary albumin to creatinine ratio. *B*, histological changes in glomeruli of db/db mice after SNHG5 knockdown shown by PAS staining (magnifications ×400). *C*, the ultrastructure of kidney for TEM (*C*1, magnifications ×8000) and fusion rate of foot process in mice (*C*2, n = 6), the *blue arrow* indicates foot process, ∗∗*p* < 0.01 *versus* sh-NC (Student's *t* test). *D*, the protein levels of nephrin and podocin relative to control in kidney of db/db mice after SNHG5 knockdown (Western blot, n = 6). *E*, the levels of SNHG5 (normalized to β-actin) and miR-26a-5p (normalized to U6) in kidney of db/db mice after SNHG5 knockdown (qPCR, n = 6). ∗∗ *p* < 0.01 *versus* sh-NC (Student's *t* test). *F*, immunohistochemistry for TRPC6 in glomeruli of db/db mice after SNHG5 knockdown (magnifications ×600). *G*, TRPC6 protein level relative to control in kidney of db/db mice after SNHG5 knockdown (Western blot, n = 6), ∗∗*p* < 0.01 *versus* sh-NC (Student's *t* test). (*D*) and (*G*) shared the same blotting sheet and reference protein. All data above are represented as means ± standard deviation. PAS, periodic acid Schiff; SNHG5, small nucleolar RNA host gene 5; TEM, transmission electron microscope; TRPC6, transient receptor potential canonical type 6.

To confirm the functional effects of SNHG5 *in vivo*, we suppressed SNHG5 function in podocytes with the SNHG5 shRNA by tail vein injection (n = 6 for each group). As expected, less albuminuria was found in db/db mice with SNHG5 knockdown (Fig. 6*A*). Meanwhile, histologic analysis revealed that glomerular hypertrophy and mesangial expansion were significantly attenuated in SNHG5 knockdown mice (Fig. 6*B*). TEM analysis revealed that foot process fusion and basement membrane thickening were markedly alleviated (Fig. 6*C*). Expression levels of nephrin and podocin were significantly increased (Fig. 6*D*). Levels of miR-26a-5p in db/db mice with SNHG5 knockdown were also increased (Fig. 6*E*) combined with decreased TRPC6 expression in podocytes (Fig. 6, *F* and *G*). These findings suggest that SNHG5 knockdown protects podocytes by modulating the miR-26a-5p/TRPC6 axis in DN.

## Discussion

DN is a therapeutic challenge in clinical practice, partly because of its unclear pathogenesis. Several lines of evidence demonstrated that podocyte dysfunction is central to the underlying pathophysiology of DN (15). Nevertheless, the mechanism of podocyte dysfunction in DN remains poorly understood. In the present study, we found that upregulated TRPC6 in injured podocytes plays an essential role in the occurrence of DN. Meanwhile, we determined miR-26a-5p as the regulator of TRPC6 in injured podocytes. Furthermore, the lncRNA SNHG5, which sponges miR-26a-5p, regulates the TRPC6 pathway.

As a terminally differentiated cell type, podocytes participate in the formation of a filtration barrier, which is critical in maintaining the physiological function of the kidney. Several lines of evidence suggest that membrane structural and signal transduction proteins in podocytes help maintain homeostasis. Among them, TRPC6, which forms a signal transduction complex with nephrin and podocin on podocytes (16), is critical to podocyte injury susceptibility in kidney diseases (17).

Studies showed that the TRPC6 channel might be a promising molecular target for developing nephroprotective agents. In the present study, we found that TRPC6 expression increased significantly in the kidney of db/db mice and HG-treated podocytes, consistent with a previous study showing that TRPC6 is upregulated in podocytes of several DM models (18, 19, 20). Two mechanisms have been proposed to explain how TRPC6 mediates podocyte injury. The activation of the ion channel induces the influx of Ca^2+^, which causes cytoskeleton rearrangement and changes the activity of downstream molecules, including NFAT (4, 20, 21). Other studies demonstrated that TRPC6 regulates the motility and detachment of podocytes by physical interaction with calpain, rather than depending on Ca^2+^ conductance (22, 23). This evidence enriches the understanding of how TRPC6 links to podocyte injury. Nevertheless, the underlying mechanism of dysregulated TRPC6 in podocytes remains largely unclear.

Studies found that miRNAs, a class of small endogenous noncoding RNAs, are potent regulators of podocyte injury in DN development (24). For example, low levels of miR-25 were detected in peripheral blood from DM patients and in kidneys of animals with type 1 and 2 DM (25), while miR-21 was upregulated in kidneys of streptozotocin-induced DM mice (26), both of which play essential roles in aggravating podocytes injury. We speculated that abnormal TRPC6 expression was regulated by specific miRNA in DN for these reasons.

In the present study, we found that miR-26a-5p is a critical regulator of TRPC6 expression. The concept that miR-26a-5p is necessary for cell growth, proliferation, differentiation, and apoptosis has gained acceptance based on studies of several pathophysiological conditions (27). Consistent with a recent study that transferred miR-26a-5p improves viability and suppresses the apoptosis of podocytes (28), we found that overexpression of miR-26a-5p protected podocytes from structural disruption by alleviating the abnormal expression of TRPC6, characterized by the amelioration of foot process effacement and recovery of podocyte structural markers (nephrin and podocin). These findings suggest that miR-26a-5p is a critical regulator of podocyte injury by targeting TRPC6. Nevertheless, the precise regulatory pathways for these effects have not been described in the setting of DN.

It is known that lncRNA participates in various pathophysiologic processes and functions. We hypothesized that specific lncRNAs precisely regulated the dysregulated miR-26a-5p/TRPC6 pathway. We performed high-throughput sequencing to identify these specific lncRNAs. Combined with *in silico* analysis, we identified an aberrantly overexpressed lncRNA SNHG5 that contains binding sites for miR-26a-5p. As expected, SNHG5 expression was markedly increased in podocytes under DN condition. Containing 524 base pairs, SNHG5 was first discovered in human B lymphoma cells by Tanaka (29). SNHG5 was involved in several tumor and nontumor diseases and is associated with clinical features, prognosis, drug sensitivity, and other factors regarding diagnosis and treatment (30, 31, 32, 33). Recently, compelling evidence indicated that upregulated SNHG5 plays a vital role in neoplastic kidney disease (34, 35). Regarding nonneoplastic kidney disease, a recent study indicated that SNHG5 expression was elevated in HK-2 cells treated with HG, accompanied with decreased cell viability, increased apoptosis, and enhanced inflammatory cytokines and oxidative stress (36). Nevertheless, the potential effects of SNHG5 in podocytes remain to be explored. In the present study, we found that SNHG5 expression was markedly increased in DN. SNHG5 levels positively correlated with ACR of db/db mice. More interestingly, attenuated podocyte injury, accompanied by restored miR-26a-5p and decreased TRPC6 expression, was found in HG-treated podocytes after SNHG5 knockdown. Meanwhile, lower levels of albuminuria and alleviated foot process fusion were observed in db/db mice with SNHG5 knockdown. Mechanically, SNHG5 knockdown protected podocytes from injury by modulating the miR-26a-5p/TRPC6 axis in DN. These findings are the first evidence for the biological function of SNHG5 in podocytes under DN condition.

Noncoding RNAs (ncRNAs, including lncRNAs, miRNAs, etc.) are essential mechanisms of epigenetics that regulate gene expression at the transcriptional and posttranscriptional levels (37). Several lines of evidence demonstrated that ncRNAs play critical roles in developing kidney diseases (9, 38). There is close crosstalk between lncRNAs and miRNAs. One of the most well-known mechanisms involves competing for endogenous RNA (39). For example, overexpression of lncRNA H19 protects the kidney against acute ischemic injury by sponging miR-30a-5p and stimulating proangiogenic signaling (40). Considering the essential role of ncRNAs, strategies for preventing or treating diseases have been proposed, including siRNAs, antisense oligonucleotides, and clustered regularly interspaced short palindromic repeats (41, 42, 43). In the present study, we also attempted to explore the potential therapeutic targets for podocyte injury *via* targeting ncRNA.

## Conclusions

**Figure 7:**
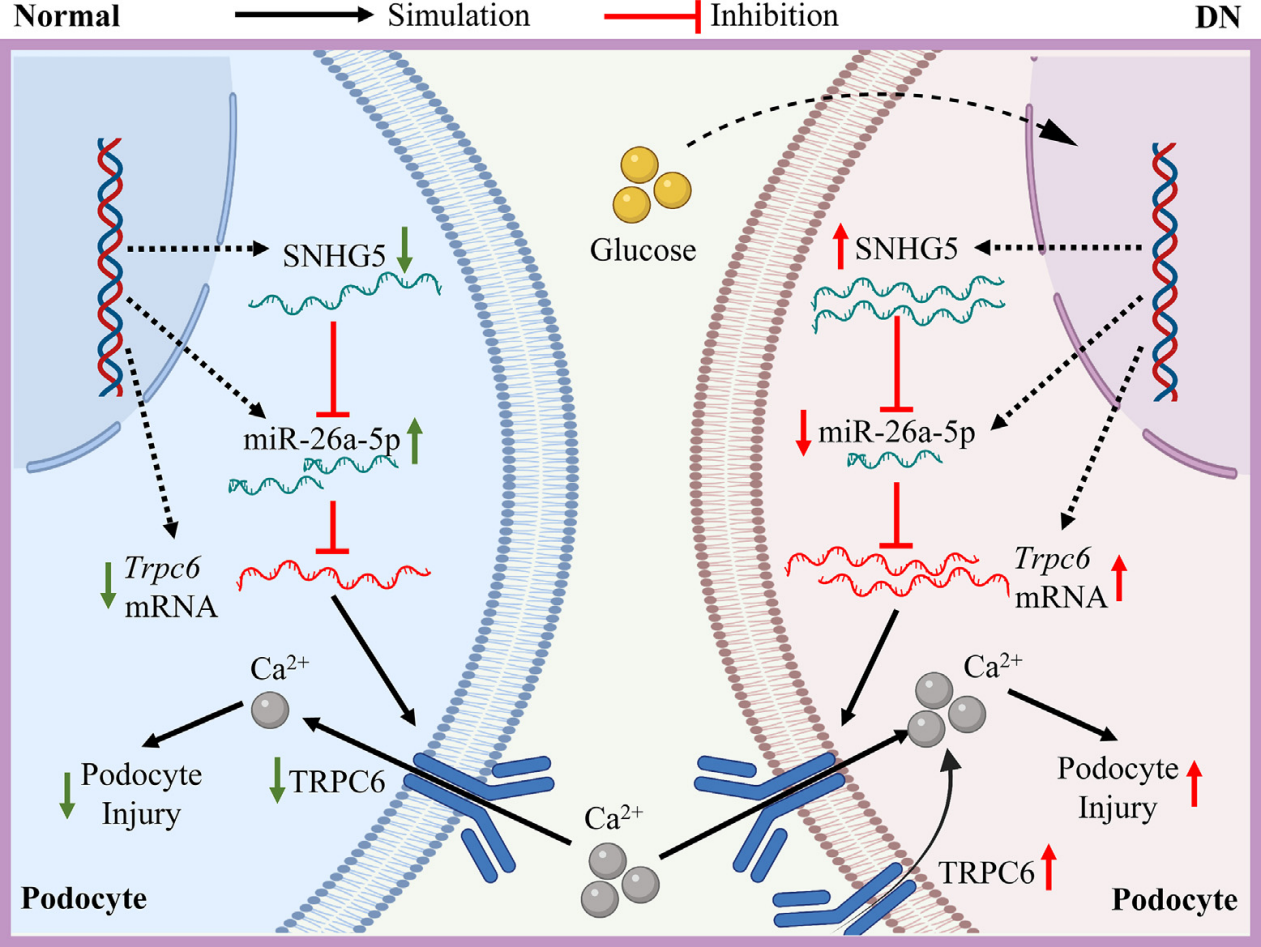
**A schematic representation describing the role of SNHG5 in podocytes in DN.** In the context of DN, SNHG5 is overexpressed with stimulation of high glucose. More SNHG5 sponges with miR-26a-5p and the expression of miR-26a-5p is decreased. *Trpc6* mRNA, a target gene of miR-26a-5p thus escapes the inhibition of miR-26a-5p and overexpressed, resulting in more Ca^2+^ influx and promotion of podocyte injury. DN, diabetic nephropathy; SNHG5, small nucleolar RNA host gene 5; TRPC6, transient receptor potential canonical type 6.

In conclusion, we discovered a precise mechanism mediating podocyte injury in DN. Upregulated SNHG5 contributes to podocyte injury by targeting the miR-26a-5p/TRPC6 pathway (Fig. 7). These findings provide insights into the mechanisms of podocyte injury and the progression of DN. Interrupting this integrated SNHG5/miR-26a-5p/TRPC6 signaling cascade represents a promising therapeutic target for DN.

## Experimental procedures

### Cell culture and transfection

Immortalized mouse podocytes (MPC5) retrieved from Shandong University were cultured in RPMI 1640 medium (Gibco) supplemented with 10% fetal bovine serum, 5.5 mM glucose, 100 mg/ml streptomycin, 100 U/ml penicillin, and 10 U/ml IFN-gamma (Sigma-Aldrich) at 33 °C in a 5% CO_2_ atmosphere incubator. For the NG group, podocytes were cultured in 5.5 mM glucose for 48 h, while for the HG and the HM groups, podocytes were exposed to high concentrations of glucose (HG, 30 mM) or mannitol (HM, 5.5 mM glucose +24.5 mM mannitol) for 48 h.

SNHG5 siRNA (si-SNHG5), miR-26a-5p mimic, and their negative control (sh-NC and mimic-NC) were designed and supplied by Gene Pharma. Podocytes were transfected with si-SNHG5 or si-NC according to the manufacturer’s protocols. The transfection efficiency was then evaluated *via* qPCR.

### Animal and treatment

A total of 30 db/db mice were divided into five groups: db/db (no interference), db/db+sh-SNHG5, db/db+sh-NC, db/db+miR-26a-5p agomir, and db/db+agomir-NC, with six mice per group. Another six db/m mice were used as normal control. The lentiviral shRNA directed against SNHG5 (sh-SNHG5), the miR-26a-5p agomir, and their negative control (sh-NC and agomir-NC) were purchased from Gene Pharma. We injected 100 μl sh-NC or sh-SNHG5 into the tail vein of db/db mice. The viral titer of lentivirus vectors was 2 × 10^13^ genomes/ml, resulting in approximately 2 × 10^12^ genomes per injection. The Ethics Committee of Affiliated Hospital of Qingdao University approved the study (Ethical Approval Number: *QYFY WZLL 26933*).

### Sample collection and assessment

Random urine samples were collected from each group of mice when they were 10 weeks old. Blood was taken from the tail vein after 12 h of fasting, after which the mice were sacrificed to obtain kidney tissues. The levels of fasting blood glucose, serum creatinine, urea nitrogen, triglycerides, total cholesterol, low-density lipoprotein cholesterol, urine creatinine, and urine albumin were measured on an automatic biochemical analyzer, and ACR was calculated (ACR = serum creatinine/urine albumin). The mice were weighed before sacrifice to obtain body weight, the left kidney was weighed after sacrifice to obtain kidney weight, and the kidney hypertrophy index (kidney weight/body weight) was calculated. Some renal cortex was stained with periodic acid Schiff (PAS), and the pathological manifestations were recorded using a light microscope; other renal cortex was stained with uranyl acetate-lead citrate, and podocyte ultrastructure was observed using a TEM. The foot process fusion rate was calculated as follows: FPR (%) = ΣLFP/ΣLBM, where ΣLFP is the total length of the fused foot process, and ΣLBM is the total length of the peripheral capillary basement membrane. The remaining kidney cortex was quickly frozen in liquid nitrogen and then transferred to a –80 °C freezer for storage.

### qPCR analysis

Levels of SNHG5, miR-26a-5p, and *Trpc6* mRNA were determined using qPCR. Total RNA of podocytes or kidneys were extracted with TRIzol reagent (Invitrogen), and reverse transcription was performed using a cDNA Reverse Transcription Kit (Takara). qPCR was performed using an SYBR Green PCR kit (Applied Biosystems). Small endogenous nucleolar U6 snRNA was used as an internal control for the normalization of miRNA and β-actin for mRNA and lncRNA. The relative expression level of genes was calculated using the (2^−ΔΔCt^) method. The sequences of primers are shown in Table 2.

**Table 2:** Primer sequences for qPCR

qPCR assays	Primers	Sequences (5′→3′)
SNHG5	Forward	CGCTTGGTTAATCCCTGACACT
	Reverse	CCAAGACAATCAGGCCTCTATC
miR-26a-5p	Forward	TCCATAAAGTAGGAAACACTACA
	Reverse	CAGTACTTTTGTGTAGTACAA
*Trpc6*	Forward	TCTCTGGTTTACGGCAGCAGA
	Reverse	GGAGCTTGGTGCCTTCAAATC
β-actin	Forward	CATCTCACCTGAAGCACCCT
	Reverse	CGGAGTCCATCACAATGCCT
U6	Forward	CTCGCTTCGGCAGCACATA
	Reverse	AACGATTCACGAATTTGCGT

### Western blot assay

Total protein of podocytes or kidneys were extracted, and protein concentration was determined using bicinchoninic acid kits (AmyJet Scientific). Proteins were separated using 10% sodium dodecyl sulfate-polyacrylamide gel electrophoresis and transferred onto polyvinylidene difluoride membranes. Membranes were blocked with 5% skim milk and incubated at 4 °C overnight after treatment with primary antibodies TRPC6 (RRID: AB_11027546, 1:1500; RRID:AB_10077516, 1:1000), nephrin (RRID: AB_11040198, 1:1000), podocin (RRID: AB_2892752, 1:1000), and β-actin (RRID: AB_10077516, 1:5000). Membranes were then incubated with the corresponding secondary antibodies (RRID:AB_2747412, 1:5000) at room temperature for 2 h after washing with phosphate-buffered saline. De-hybridization were performed with the Western blot stripping Buffer (21059, Thermo Scientific) to reuse the same blots for other molecules detection. The protein bands were visualized using an ECL reagent (Thermo Scientific) and quantified using ImageJ software (NIH).

### Immunohistochemistry stain

After baking, deparaffinization, and rehydration, kidney sections were blocked with 10% calf serum in PBS. Then, they were incubated with TRPC6 primary antibody (RRID: AB_11027546, 1:1000) at 4 °C overnight, followed by PBS washing and incubation with biotin-conjugated goat anti-rabbit IgG (RRID:AB_2864333) for 30 min at room temperature. Immunoreactivity was detected using diaminobenzidine reagent (Solarbio). The sections were then stained with hematoxylin and covered with neutral resin. Images were obtained with an optical microscope (Leica Microsystems).

### FISH

Gene Pharma designed the SNHG5 and miR-26a-5p FISH probes. The subcellular localization of SNHG5 and miR-26a-5p were assayed according to the manufacturer’s protocol. Sections were removed from 4% paraformaldehyde and permeabilized with Triton X-100, followed by a hybridization buffer containing probe. Sections were finally costained with DAPI and observed using fluorescence microscopy.

### Luciferase activity assay

The wildtype and mutated SNHG5 and *Trpc6 mRNA* were subcloned into a pGL3 vector (Promega). HEK-293 tool cells were cotransfected with the plasmid constructed with pGL3-SNHG5 or pGL3-SNHG5-mut (pGL3-*Trpc6* or pGL3-*Trpc6*-mut) and miR-26a-5p mimics or mimic-NC using Lipofectamine 2000 (Invitrogen). After 48 h of incubation, firefly luciferase and Renilla luciferase activity were measured using the Dual-Luciferase Reporter Assay System (Promega), and the relative luciferase activity was calculated.

### RIP assay

The RIP assay was performed using an Imprint RNA Immunoprecipitation Kit (Sigma-Aldrich). Podocytes transfected with SNHG5 were harvested and solubilized in RIP buffer. All decomposition products of podocytes were incubated with magnetic beads and anti-IgG antibody or anti-Ago2 antibody (Abcam) overnight at 4 °C. qPCR was used to measure the level of SNHG5 after purification of the coprecipitated RNAs.

### RNA pull-down assay

Podocytes transfected with Biotinylated miR-26a-5p or Bio-NC were harvested and lysed. Cell lysates were cultivated with streptavidin-coupled magnetic beads (Invitrogen) to generate probe-coated beads and incubated overnight. Total RNA was extracted, and SNHG5 expression was determined using qPCR.

### Statistical analysis

The data were expressed as means ± standard deviations. Complete data with a normal distribution were analyzed using the Student's *t* test (between two groups) or one-way analysis of variance (for several groups). Correlation analysis was performed using *Pearson* analysis. SPSS 25.0 (SPSS Inc) was used for data analysis, and *p*-value <0.05 indicated that the difference was statistically significant.

## Data availability

The data used to support the findings of this study are contained within this manuscript.

## Conflict of interest

The authors declare no conflict of interest with the contents of the article.
